# A post-authorisation survey to analyse the perioperative teicoplanin plasma concentrations in adult patients with chronic bone sepsis, who received loading doses of 12 mg/kg 12-hourly for 48 hours followed by 12 mg/kg once daily

**DOI:** 10.1186/cc10676

**Published:** 2012-03-20

**Authors:** AJ Brink, G Richards, C Lautenbach, N Rapeport, V Schillack, J Roberts, J Lipman

**Affiliations:** 1 Milpark Hospital, Johannesburg, South Africa; 2 University of Witwatersrand, Johannesburg, South Africa; 3 Ampath National Referral Laboratory, Pretoria, South Africa; 4 University of Queensland, Brisbane, Australia

## Introduction

To rapidly achieve teicoplanin trough (C_min_) concentrations ≥20 mg/l suggested for sternal sepsis, loading doses higher than 6 mg/kg 12-hourly might be warranted [1].

## Methods

Patients (*n *= 10) with deep-seated Gram-positive infections were enrolled perioperatively. During the first 4 days of therapy teicoplanin loading doses of 12 mg/kg 12-hourly were administered for 48 hours and 12 mg/kg once daily thereafter. Surgical debridement was performed on D3. Samples were collected 15 minutes before and 30 minutes and 120 minutes after each teicoplanin administration. Total and unbound teicoplanin levels were determined using HPLC.

## Results

All patients had hypoalbuminemia (mean 20.2 g/l). The SS PK parameters of teicoplanin are described in Table 1. On D3 the median total and free C_min _were 14.66 (8.93 to 19.66) and 3.09 (0.0 to 6.4) mg/l, respectively. In a multivariate logistic regression model, total teicoplanin concentrations (*P *= 0.174) and serum creatinine concentration (*P *= 0.034) did not impact significantly on free teicoplanin levels whereas, in contrast, albumin concentration did (OR 0.120, 95% CI 0.078 to 0.161, *P *< 0.001).

**Table 1:** Steady-state pharmacokinetic parameters of teicoplanin

	Total	Free
C_max_	20.1	2.6
C_min_	6.7	2.3
AUC	137.9	28.6
CL	7.0	33.5
Vz	174.1	196.6

## Conclusion

The levels achieved on D3 in this study are similar to those achieved by Mimoz and colleagues using the same dosing schedule in ICU patients with VAP [2]. Only hypoalbuminemia impacted on the free levels of teicoplanin in this setting. High teicoplanin loading doses of 12 mg/kg 12-hourly should probably be extended beyond 48 hours, before major elective surgery for chronic bone sepsis.
